# Making Sense of Point‐Of‐Care Testing for the Acute Management of Respiratory Tract Infections in Young Children: Caregiver Experiences in Sweden

**DOI:** 10.1111/apa.70705

**Published:** 2026-07-21

**Authors:** Helle Mølsted Alvesson, Ingela Zenk, Reza Rasti, Tobias Alfvén

**Affiliations:** ^1^ Department of Global Public Health Karolinska Institutet Stockholm Sweden; ^2^ Sachs´ Children and Youth Hospital Stockholm Sweden; ^3^ Sparta Children's Clinic Lund Sweden

**Keywords:** interviews, paediatric emergency department, point‐of‐care testing, respiratory tract infections, Sweden

## Abstract

**Aim:**

This study examined how parents perceive and experience point‐of‐care testing in a paediatric emergency department and how these tests relate to the overall diagnostic process. Parents sought care for their children due to suspected respiratory tract infections.

**Methods:**

Twenty parents participated in semi‐structured interviews. These interviews were analysed using content analysis and guided by the theory of symbolic interactionism.

**Results:**

Parents considered testing an important part of the consultation and found it easy for the child to undergo. Performing tests was a tangible way for parents to understand the progress of care. When tests were not performed, parents expected a reason for their omission. The tests were viewed as symbolic markers that the diagnostic process was advancing and as proof to parents that visiting the paediatric emergency department was the correct choice.

**Conclusions:**

Parents interpreted point‐of‐care testing as a potent medical and symbolic tool to discover or rule out serious diseases. To prevent the risk of confusing test results with a diagnostic label, clear communication between caregivers and providers can alleviate uncertainty.

## Summary

- Parents found point‐of‐care testing to be a potent technology in extracting biological information from the child's body, in contrast to only looking at the child.
- Point‐of‐care tests reinforced the legitimacy of emergency care, symbolised the provider–parent relationship, and shaped parents' self‐image as responsible caregivers.
- To avoid parents mistaking point‐of‐care test results for definitive diagnoses, paediatric emergency departments must clearly explain the rationale behind medical testing.

**Abbreviations**
PED: pediatric emergency department
POCT: point‐of‐care test
POC‐testing: point of care testing
RSV: respiratory syncytial virus
RTI: respiratory tract infection

## 1 Introduction

User understanding of diagnostic testing when seeking emergency health care is crucial for the quality of care and patients seeking care at the appropriate facility level. The process of reaching a diagnosis is fundamental to modern biomedicine and can be analysed with the same rigour as the categorisation of disease [1]. Diagnosing respiratory tract infections (RTIs) is complex, and evidence from primary health care shows that clinicians can prioritise treatment decisions over establishing a precise diagnosis [2]. While the categorisation of disease is increasingly formalised through updated international classification standards, the processes through which a diagnosis is set are less formalised [3]. The relationship between the diagnosis of paediatric RTIs and the technical processes employed to inform these diagnoses has evolved rapidly in response to advancements in medical testing methodologies [4].

Globally, RTIs are a leading cause of mortality in children under 5 years and a frequent cause for attending paediatric emergency departments (PED) [5]. However, the majority of RTIs are self‐limiting and, in most cases, manageable at home. Furthermore, although most RTIs are viral in aetiology, antibiotics continue to be over‐prescribed in paediatric care worldwide [6], contributing to an increased global burden of antibiotic resistance [7]. One strategy to reduce demand for and over‐prescription of antibiotics is to promote point‐of‐care tests (POCTs) as a diagnostic tool. In the case of paediatric RTI, these short‐time‐to‐results tests act as a useful tool to corroborate the doctor's findings via the measurement of the level of C‐reactive protein in the blood [4].

The anthropology of medical testing is a growing field in which POCTs are analysed as involving social and cultural processes [1]. The focus is on experiences of the testing process rather than the specific diagnostic technology. This includes ideas on user perceptions of receiving results during the consultation and experiences of convenience, accessibility, and reassurance [1].

Studies on POCTs have highlighted how values and assumptions are ascribed to this type of testing [8, 9]. Social relations in healthcare provision are transformed, and the tests are credited with new meaning by users [10]. In this paper, we will contribute to the understanding of POCTs through adopting Blumer's version of symbolic interactionism as a theoretical lens [11]. Drawing on three core ideas about how people relate to objects through meaning, interpretation, and interaction, we have turned to symbolic interactionism to understand the POCT‐related practices at an emergency department. Symbolic interactionism emphasises how individuals create meaning through interaction, how they shape and present their identities, and how they define situations with others [11]. Our actions follow from the way we have interpreted and defined the situation.

The objective is to understand the different meanings caregivers assign to POCTs. The meaning is not inherent in a test or other medical object but is created through interpretative interactions between people and the object. Blumer stated that ‘meanings are handled in, and modified through, an interpretative process used by the person dealing with the things he encounters’ [11, p. 2]. This suggests that the use of technology can reshape the interpretive processes and social relations involved [12], such as during a consultation at a PED.

Medical consultations are situations in which patients and providers assess their roles and relationships. When understanding social life as produced through the use of symbols, people are viewed as self‐reflective agents who modify their social behaviour according to situation and position [13].

From several studies, we know that provision of diagnostic testing is related to care seekers' satisfaction [4, 14]. Targeted POC testing in PED for streptococci and HIV is feasible and acceptable when communicated well [15, 16]. POCTs became popular during the COVID‐19 pandemic, but the pandemic also highlighted the uneven access and the commoditisation of tests [17]. A few studies exist on parents' satisfaction regarding communication and child‐friendly care in a PED setting [18, 19], but none concerning experiences with POC testing in emergency care. We found one study in a primary health care setting with adults seeking care for respiratory tract infections in Sweden [20], where it was observed that the decision to administer the C‐reactive protein POCT was interactionally motivated and organised. Doctors ordered testing even when the outcome of the physical examination was assessed as normal with no infection, suggesting that medical indications were not the only reasons for testing. The study also showed that doctors routinely explained their reasons for ordering the test; when they did not, patients tended to question the purpose of the test [20].

POCTs share many features, that is, they are administered close to the patient, results can be available rapidly and they can influence decision‐making of next steps during the same patient‐provider encounter. We are interested in the perceived social function of medical tests and aim to understand how caregivers experience the process of POCT and how test taking shapes caregiver satisfaction during a visit to a paediatric emergency room.

## 2 Method

The reporting of this study has been guided by the values‐based guidelines for qualitative studies developed by Braun & Clarke [21].

### 2.1 Study Design and Setting

Semi‐structured interviews were conducted with parents seeking care for their young children at the paediatric emergency department of a public tertiary care hospital. It is one of the largest paediatric in‐ and outpatient hospitals in Stockholm, Sweden, visited annually by around 30 000 patients aged 0–18, with approximately 10% of patients below the age of five seeking care for respiratory symptoms. At the time of the study, the most used POCTs for children with suspected RTIs were those testing for C‐reactive protein (measured in blood) and respiratory syncytial virus (RSV) (measured via nasopharyngeal swab), and sometimes also capillary blood gases. Additional tests were acid–base, Strep A and urine tests. The use of POCTs was not guided by specific guidelines at the emergency department where the study was conducted. Their use was at the discretion of each clinician. At the time of the study, the availability of POCTs differed between the three tertiary PEDs in Stockholm. In the study setting, POCTs for RSV and Influenza A/B were readily available and used when deemed necessary. In contrast, in another Stockholm tertiary PED, POCT for RSV was only used for patients with respiratory symptoms being admitted, to support cohort care.

Parents attending the PED with children younger than 5 years old with respiratory symptoms were eligible to participate in the study. Parents of children who were admitted to the hospital were excluded, based on the assumption that the use of POCTs would be of secondary importance when a child was ill enough to require hospitalisation. It was also deemed inappropriate to ask parents of hospitalised children to participate in interviews during the care period. Furthermore, conducting interviews after discharge was considered unhelpful, as the parents' impressions and concerns related to the hospitalisation would likely overshadow their recollection of whether rapid tests had been performed in the emergency department.

A research nurse (IZ), who was not involved in the care of the children, approached parents when they were leaving the hospital with oral and written study information and collected signed consent forms from participants. Out of a total of 22 eligible parents during the working hours of IZ during the period January–April 2017, 20 took part in the study.

### 2.2 Data Collection and Analysis

A draft interview guide was prepared based on literature on paediatric emergency care and user perspectives on medical devices, with a focus on POCTs (See Appendix S1). The guide was structured chronologically and started with questions about reasons for visiting the PED and experiences of the care received, with emphasis on the diagnostic process, including the use of POCTs. During the interviews, IZ sought to strike a balance between exploring caregivers' perceptions of the different POCTs and ensuring that they did not feel their medical knowledge was being tested.

The interviews focused on this most recent visit to the emergency department. For three of the children, they had been to the emergency department two times during the previous 2–3 days. Both consultations were discussed in the interview.

Two telephone and two in‐person pilot interviews were initially conducted. During this process, the draft interview guide was revised to ensure comprehensibility, and it was decided to conduct in‐person interviews only due to better rapport building with the parents. A few days after the consent form was signed, IZ contacted the parents to confirm their participation and conduct the interview. The interviews were conducted in Swedish and lasted 35–90 min, with an average of 58 min. They were audio recorded and transcribed verbatim by IZ.

Table 1 describes the main characteristics of the participants and their children. The parent attending the PED was in most cases the mother (*n* = 13); one father attended with one child, while both parents attended in 6 cases. If both parents attended the PED with the child, it was their choice if one or both should be interviewed. If both parents participated, they were interviewed together. In 16 cases, the interviewee was the mother, in two cases it was the father, and in two cases both parents were present.

**TABLE 1:** Characteristics of the participating parents and their children with suspected respiratory tract infection.

Characteristic	Number of interviews (*N* = 20 by 22 parents)
Child's age (months)	
0–2	2
> 2–6	6
> 6–12	9
> 12–18	2
> 18–59	1
Birth order of the infant	
1st	8
2nd	9
3rd	2
4th	1
Sex of child	
Female	7
Male	13
Parent's age	
20–29	7
30–39	14
> 40	1
Parent's level of education	
Secondary school	2
Upper secondary school	6
Higher degree	14

Data was analysed using qualitative content analysis according to Krippendorf [22]. After the first line‐by‐line coding in NVIVO 11.4.0, clusters of data were developed through mind maps. From this iterative process, new codes were developed and discussed by IZ and HMA, and later with all authors in several rounds. With time and through author discussions, the relevance of using the theory of symbolic interactionism became clear. One example of the need of theory was our initial difficulty in understanding why parents wished for POCTs even when they did not receive the results promptly—or at all. Symbolic interactionism provided a tool to understand how parents attached significance to the testing and interpreted the medical practices in different ways than intended by the providers [11].

### 2.3 Ethics

Since their child was sick at the time parents were approached, they were asked to reconfirm their participation before the interview was conducted. At the hospital, it was explained to parents that study participation would not have any influence on the child's care. The study was approved by the Swedish Ethical Review Authority (ref. 2016/2296‐31).

## 3 Results

The analysis resulted in 4 themes: (i) Parents lose and try to regain control of care in periods of uncertainty; (ii) POCTs are representations of clinical effectiveness; (iii) POCTs reach knowledge hidden inside the body of the child; (iv) Testing is validation of being a good parent (See Figure 1 below). No differences between parents in terms of demographic characteristics were identified in the analysis, and we refer to the participants as one group.

**FIGURE 1:**
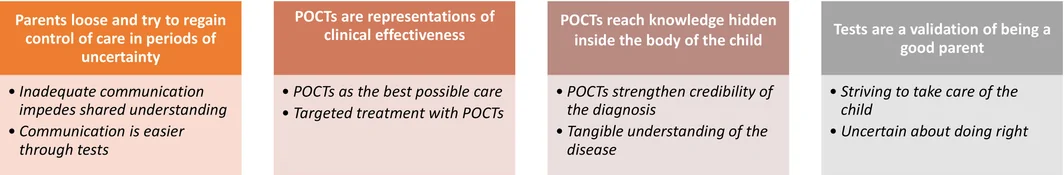
Results of content analysis presented in four themes with sub‐themes.

### 3.1 Parents Lose and Try to Regain Control of Care in Periods of Uncertainty

Parents were seeking help with the care of their child. Upon arrival at the PED this implied that they expected to hand over control of the care of their child to the emergency healthcare providers (referred to as providers in the remainder of the paper) during the subsequent consultations. They tried to regain control through talking about test results.

#### 3.1.1 Inadequate Communication Impedes Shared Understanding

Since the PED provides specialised care, parents had an expectation before the visit that medical tests would be performed, but this was not always the case. Parents were surprised when they asked for testing to be performed and were informed that *‘that is not necessary’* without being given any reason for this assessment. Lack of testing led to uncertainty about why the provider ruled out testing.

… I questioned it, since we came here to have some tests taken … But he (baby) looks alright. [They said] it is not needed [to test] and then there was no more information given. Then she (the doctor) disappeared. (Mother of child 2 months)

When POCTs were performed, parents reported that they were not always informed about why tests were taken, what they might indicate, or how they would be performed on the child.

The first time they didn't tell me anything, tests were just taken and then they left … I wasn't told what test they took or how long it would take … The second time they told me they took a blood sample from the foot, but not why. The difference was that the first time they took an RSV test, and the second time blood tests. I have no clue why they did different things, and nobody gave any information either … (Mother of child 3 months)

Other parents described not having the courage to ask questions, being insecure about what to ask about, and feeling ignorant about which tests were available. Parents tried to refrain from bothering staff for fear of being seen as wasting staff time.

Although parents expressed that they trusted that the provider would have told them if something had been abnormal, they expressed an interest in knowing what had been ruled out or what the tests meant. Sometimes, they were told at discharge ‘*it's nothing*’, which was considered a very confusing and frustrating test result.

… it is so stressful, and then simply: no, it's nothing … there is nothing wrong with her. Everything looks fine, but oh God what CAN it be then, if there is nothing wrong … (Mother of child 4 months)

#### 3.1.2 Communication Is Easier When Using Tests

POCTs were regarded by parents as a clear sign that the process was moving forward, leaving them with a sense of control, knowing that they were waiting for the results. If the parents believed that there was progress in the diagnostic process, the actual waiting time seemed to be of less importance. Parents felt that asking for test results was a concrete and acceptable reason for reaching out to the staff and informing them that they were still waiting, without feeling that they were interfering.

### 3.2 POCTs Are Representations of Clinical Effectiveness

#### 3.2.1 POCTs as Best Possible Care

Parents were not reassured by a physical examination alone. POCT in combination with a thorough physical examination that resulted in an explanation of the child's symptoms was considered to be the best possible care. This meant that the parents could return home with a feeling of regained control and reassured that they had done everything in their power to ensure the child would recover, with or without medical treatment.

… They give you a quick answer to what if something is wrong … actually, I am really positive about that … I am positive about any kind of test that can give you insight. I'd rather have tests than be speculating … it can be all sorts of things … and especially for me, who does not have a clue about what it could be … (Father of child 11 months)

The wish to understand the grounds on which the provider decided not to verify the physical examination using POCTs was strong and resulted in questions such as, *‘how can you know just by looking at a child?’*. Parents found it difficult to understand why tests were not used.

For my benefit as a parent and also for my child, I find it rather important that tests are taken in order to exclude and evaluate. Sometimes you think it is nothing, no it doesn't look serious, but then they take tests indicating that there is actually something after all … (Mother of child 2 months)

Parents saw POCTs as a diagnostic solution due to their rapid results.

I spontaneously feel that rapid tests are perfect for an emergency department. When you go to the emergency department because you couldn't get a handle on the fever, then the rapid tests are great, I think. The more rapid tests you can take, the better it is, to rule out a lot of other stupid things, as well. (Mother of child six months).

A couple had sought care twice during the same week with their 11‐month‐old son and they clarify the importance of getting answers quickly:

… I think it (POCT) is good because you sit there for so long and you want to know quickly … Now the sed rate is on this and probably it is this and, well, no. I think it's good that you get to know quickly. (Couple with 11 months old)

Paradoxically, parents had in most cases waited several hours before receiving the test results—if at all.

#### 3.2.2 Targeted Treatment With POCTs

POCT confirmation of a non‐severe infection reassured parents that the child would recover without hospital admission. However, parents described a fear that the child might have RSV. The uncertainty of not testing for it caused anxiety, even though they knew that there was no treatment for the infection.

… providing the right treatment, if there is a possibility to take a test … then surely it must be better to do it and base treatment on test results rather than just give treatment based upon … visual things … (Mother of child 13 months)

It should be noted that RSV was the only virus that parents reported and this was the case for the parents to young infants. On receiving a viral diagnosis, parents valued advice on how to relieve the symptoms in cases where no medication was recommended. Most parents were aware that antibiotics do not have an effect on all types of infections and that it is not necessary to treat a virus with medication other than antipyretics, such as paracetamol or ibuprofen. Some had understood from the PED that an elevated C‐reactive protein test contributed to the decision to prescribe antibiotics. In fact, parents wished to avoid unnecessary medical treatment, expressing gratitude for not having to treat their child with antibiotics if not necessary. Parents expressed concern about the growing problem of antibiotic resistance, and it was mentioned that if the child were to receive antibiotics, parents preferred POCT confirmation.

… I mean, I would like it to be testing‐based … if it is possible …, identifying when antibiotics really are needed, then you wouldn't have to administer medication in vain … (Mother of child 13 months)

### 3.3 POCTs Reach Knowledge Hidden Inside the Body of the Child

All parents reported having experience with diagnostic testing of their child, either from this visit or from previous ones. These included POCTs or laboratory testing, and occasionally both. The two most mentioned tests were those analysing blood for levels of C‐reactive protein and tests identifying RSV in nasal mucus. There were only two situations in which parents said that they would accept a diagnosis without POC testing: if the physician gave a clear diagnosis, such as an ear infection, or if treatment given at the PED showed immediate effect.

#### 3.3.1 POCTs Strengthen the Credibility of the Diagnosis

Parents viewed POCTs as important tools for confirming the diagnosis proposed by the physician, since they established whether the child's symptoms were due to a viral or bacterial infection. They also indicated the severity of infection and ruled out worst‐case scenarios feared by the parents.

If they take tests, you really get it black and white, that this is what it is. And the more tests you take, the better it is, it feels like. Then you can rule out a lot of other things … (Father of child 11 months)

It was also noted that although a diagnosis without testing might be sufficient, tests provided additional reassurance and helped physicians rule out the possibility of serious infections. The tests performed were often viewed as part of routine care, and parents expressed surprise and dissatisfaction when no tests were initiated. Young infants were perceived to be more vulnerable as they could deteriorate faster, and the parents expressed concern that they themselves or the physician would miss important signs if no tests were performed.

#### 3.3.2 Tangible Understanding of the Disease

A measurable, numerical test result was regarded by parents as a reliable way for the provider to assess the seriousness of the child's condition. Furthermore, if parents were concerned that the child's condition had not improved, they could return to the PED and compare new and previous test results.

Returning again to the PED that was a bit so and so; maybe it was a little unnecessary but maybe not because I am calmer now after having a CRP test taken and I can see that it is moving in the right direction—it is probably a virus and I know we can continue with paracetamol and ibuprofen. (Mother of child 9 months)

### 3.4 Testing Is a Validation of Being a Good Parent

#### 3.4.1 Striving to Take Care of the Child

The need and the wish to do what was presumed to be best for the child was an important part of the decision to visit the PED. Waiting time was expected but was easier when information about the next steps was given. The provision of information also encouraged parents to take a more active part in monitoring and comforting the child. Parents did not expect to receive information about the actual waiting time, but rather about what the next steps would be.

… first we were sitting and waiting, but once we had seen the doctor I kind of knew what to expect … there were long waiting times as well after that … but then you knew; now she had examined (the child) and that the next thing to expect was for someone entering and letting my child inhale saline and after that the doctor should return eventually to listen to his chest again. It means you know the expected process and that to me is really good information … (Mother of child 11 months)

#### 3.4.2 Uncertain About Doing the Right Thing

From the parents' perspective, when tests were performed on the child, it meant that the attending physician took their child's symptoms seriously and acknowledged their concerns as caregivers. They were worried that the visit might be judged as inappropriate or unnecessary and expressed fear of not being listened to or criticised by the providers. They were afraid of being dismissed as overreacting or wasting valuable staff time, resulting in a feeling that they perhaps should not have come at all. Having POCTs performed was an important confirmation for parents that the decision to consult the PED was the right thing to do after all, and that the visit was appropriate in the eyes of providers.

## 4 Discussion

Parents' interpretation of the overlap of medical, social, and symbolic practices at a PED can help us understand how parents' perspectives on medical testing fit into the diagnostic process. Parents seeking care for their child with RTI symptoms perceived POCT as reassuring in a stressful situation. It was understood to strengthen the credibility of the diagnosis, rule out serious conditions, and provide reassurance that consulting the PED was the appropriate course of action. It should be noted that this study focused on families where the child was not admitted to the hospital. Parents of children who were admitted to the hospital may not see POCTs as tools for ruling out serious disease.

Parents seek to establish relationships with the providers, but since communication often tends to be unsatisfactory, feelings of stress and uncertainty are commonly experienced. Previous research has shown that patients seek to regain control over the uncertainty of illness by gaining knowledge [10]. Several studies have pinpointed the importance of communication between parents and providers and how a lack thereof often results in a lack of satisfaction with the care provided [18, 19].

Using symbolic interactionism as a theoretical lens, we were able to understand why lack of testing was perceived as withholding the best care, parents' concerns about the impression they made on healthcare providers, and their appreciation of the use of POCT, even when they were not provided with the results of these tests. POCTs were perceived as an important symbolic action that demonstrated that the physician was taking the child's symptoms seriously, that the parental concern for their child's health was recognised, and that the visit was judged to be appropriate and necessary. This validation was important to parents. This finding is in line with a study on patient behaviour when seeking emergency care [23]. It was found that the initial problem‐presentation is the patients' main opportunity to present and articulate their own agenda before the provider's questioning takes over. Patients must present themselves as legitimately sick to demonstrate that it was responsible to seek care. Research also shows that fear of criticism by professionals, in terms of illness not being serious enough for seeking emergency care, reduces parental self‐esteem and self‐efficacy and may also result in delayed appointment seeking in the future [24, 25]. Providers should be aware of parents' strategies to avoid criticism when children are sick, and that parents assume that care providers assess and judge them as caregivers of the children.

POCTs played an important role in consultations by making a part of the diagnostic process concrete and measurable and therefore acceptable to parents. In line with this, Peräkylä [26] found in a study in Finland that doctors adapted their ways of delivering the diagnosis to the availability of evidence. They did not portray their diagnosis as direct representations of the bodily reality but pointed to sources of evidence including test results (p. 219). In our study, when antibiotics were to be prescribed, parents preferred having the presence of bacterial infection established by POCTs. This is in line with another study in Sweden describing parental agreement to prescription of antibiotics only when deemed necessary [27]. In other studies, contradictory findings suggest that physicians believe that parents expect antibiotics when consulting with children suffering from RTI symptoms [28, 29, 30].

Trust in medical testing was high, and use of medicines or advanced technology was considered by caregivers to be an important part of the consultation, expected to help in recovery. Parents in a UK primary care setting described how they sometimes found a viral diagnosis trivialising when children presented with RTIs [29] and judged the credibility of viral diagnosis based on their perception of the physician's ability to communicate and the thoroughness of the physical examination. In our study, a viral diagnosis was acceptable if parents felt that they had been listened to and a thorough physical examination had been performed and validated by a POCT. Better communication between parents and health care professionals has previously been discussed as a way to facilitate acceptance of a viral diagnosis and thereby reduce antibiotic prescription [18, 19]. Testing without a clinical indication is expensive and poses a work burden on the providers. This point was addressed in our study with providers at the same emergency department [9]. Implementation of POCTs into clinical guidelines would be favourable as means to elevate quality of care and avoid over‐ or under‐use.

In this study, parents described the importance of understanding how their child was being treated but did not receive information about why the POCTs were performed and what their results meant. Wanzer et al. [31] found that parents do not necessarily understand diagnosis or treatment but judge the quality of care their children receive based on the quantity and quality of communication from healthcare professionals. However, further research shows that clinicians cite time pressure as a major limiting factor with regard to informing or educating parents [28, 29, 30]. To prevent the risk of confusing test results with a diagnostic label, communication between patients and providers becomes increasingly important with the use of diversified diagnostic practices.

The methodological limitations of this study were that it was conducted at a single hospital, and participants were relatively well‐educated compared to the general population in Stockholm. Results may therefore not be transferable to PEDs attended by different socioeconomic groups of parents. We considered there to be more risks than benefits in conducting the interviews while the parents were at the PED, and recall bias during the home‐based interviews cannot be excluded. Nonetheless, the narrative format of the interview helped the parents to remember details of the consultations. There is a risk of social desirability bias. However, since the topic was not considered to be sensitive, and the participants were assured of anonymity, this was not considered to be a high risk.

Throughout the research process the sample size was evaluated to reach information power as described by Malterud et al. [32]. The study aim was kept narrow; participants were selected due to their specific care‐seeking behaviour and the theory on symbolic interpretation of care practices was applied. The dialogue was strong since the data collector had background knowledge on the subject, and trust was quickly established during interviews.

## 5 Conclusion

Use of POCTs during PED consultations was a concrete way for parents to understand the severity of the child's illness and to monitor the provision of care. The popularity of the POCTs was linked to a notion of diagnostic certainty and, more specifically, an ability to identify or rule out danger inside the body of their child. POCTs also had symbolic value in confirming the validity of parents seeking care from a PED and reassuring them that their child had received the best available care. Yet, parents expressed a need for improvement in communication surrounding the use of POCTs and the interpretation of their results.

## Author Contributions

**Ingela Zenk:** conceptualization, methodology, formal analysis, project administration, writing – original draft, writing – review and editing, data curation, software. **Reza Rasti:** conceptualization, methodology, writing – review and editing, supervision. **Tobias Alfvén:** conceptualization, methodology, supervision, resources, writing – review and editing, funding acquisition. **Helle Mølsted Alvesson:** conceptualization, methodology, writing – original draft, writing – review and editing, formal analysis, data curation, supervision, validation, software.

## Funding

This work was supported by ALF funding (Grants 20150503 and 20180292) and Foundation Frimurare Barnhuset in Stockholm (Grants 2017 and 2019).

## Conflicts of Interest

The authors declare no conflicts of interest.

## Supporting information


**Appendix S1:** Interview guide—caregiver interviews.

## Data Availability

The data that support the findings of this study are available on request from the corresponding author. The data are not publicly available due to privacy or ethical restrictions.
