# Supplementary material for: Making Sense of Point‐Of‐Care Testing for the Acute Management of Respiratory Tract Infections in Young Children: Caregiver Experiences in Sweden
Source: Acta Paediatr. 2026 Jul 21;115(12):2872–9. doi: 10.1111/apa.70705 (PMC13747701; doi:10.1111/apa.70705)
Supplement: Supplementary file 1 [file APA-115-2872-s001.docx]

**Appendix S1- Interview guide – caregiver interviews**

The guide has been translated from Swedish to English. Probes were added to the questions based on the initial responses of the participant.

Questions

How is the child doing now?

Describe the child's symptoms and explain what the process looked like before you sought treatment.

Can you describe what was decisive for you to make the decision to go to the emergency room?

Tell us about your experience in the emergency room before you saw the doctor?

The medical examination **-** what, in your opinion, is important during a medical examination in the emergency room of your sick child?

What does it mean to you that the healthcare professionals explain what they are doing – what happens next?

What does it mean to you that tests are taken from your child at this emergency visit?

IF TESTING WAS CARRIED OUT

Tell us about the testing.

Tell us about your thoughts on the test result.

Tell us what it means to you to know/not to know which virus your child was affected by.

What is your position on the use of rapid tests and laboratory-based analyses on children?

How did you experience the possible treatment that your child received in the emergency room?

Tell us how your expectations for the visit matched reality

NO TESTING

Tell us what it means to you that no tests were made on your child at the visit in question

What diagnosis did the child receive?

Tell us what it meant to you not to know which virus your child was affected by?

What is your position on the use of rapid tests and laboratory-based analyses on your child?

How did you experience the possible treatment that your child received in the emergency room after going home?

Tell us how your expectations of the visit matched reality

CONCLUSION (all)

What were you satisfied with/less satisfied with during the visit

What advice did you receive?

Tell us about your thoughts/feelings when you came home from the emergency room

Is there anything you'd like to add that we haven't talked about?

Thank you for participating!
